# UCE: A uracil excision (USER™)-based toolbox for transformation of cereals

**DOI:** 10.1186/1746-4811-6-15

**Published:** 2010-06-10

**Authors:** Kim H Hebelstrup, Michael W Christiansen, Massimiliano Carciofi, Birgitte Tauris, Henrik Brinch-Pedersen, Preben B Holm

**Affiliations:** 1 Aarhus University, Faculty of Agricultural Sciences, Department of Genetics and Biotechnology, Forsøgsvej 1, DK-4200 Slagelse, Denmark

## Abstract

**Background:**

Cloning of gene casettes and other DNA sequences into the conventional vectors for biolistic or *Agrobacterium*-mediated transformation is hampered by a limited amount of unique restriction sites and by the difficulties often encountered when ligating small single strand DNA overhangs. These problems are obviated by "The Uracil Specific Excision Reagent (USER™)" technology (New England Biolabs) which thus offers a new and very time-efficient method for engineering of big and complex plasmids.

**Results:**

By application of the USER™ system, we engineered a collection of binary vectors, termed UCE (USER cereal), ready for use in cloning of complex constructs into the T-DNA. A series of the vectors were tested and shown to perform successfully in *Agrobacterium*-mediated transformation of barley (*Hordeum vulgare *L.) as well as in biolistic transformation of endosperm cells conferring transient expression.

**Conclusions:**

The USER™ technology is very well suited for generating a toolbox of vectors for transformation and it opens an opportunity to engineer complex vectors, where several genetic elements of different origin are combined in a single cloning reaction.

## Background

The small-grained cereals barley and wheat are major global sources of food, feed and fibre. Like for other cereals well-functioning transformation systems have been developed based on *Agrobacterium tumefaciens *or biolistics. As the two species are very closely related genetically, barley is often used as a model for wheat and many transformation constructs can be used interchangeably. A number of vector systems for transformation are available for both species and toolkits of transformation vectors are offered such as the Gateway compatible pGreen vectors from BRACT (Biological Resources for Arable Crop Transformation) (http://www.bract.org/ and http://www.pgreen.ac.uk/).

While the traditional vectors have served successfully for a number of transformation experiments they do have a number of short-comings. Cloning has to be performed in a multiple-cloning site (MCS) with a limited amount of unique or dual represented endonuclease recognition sites. The fragment to be ligated into an MCS must not contain the endonuclease recognition site used for opening the plasmid which can complicate the insertion of bigger fragments. Cloning of two fragments into the MCS during a single ligation reaction is often difficult or impossible. PCR product fusions are often used to combine two or more DNA fragments, which can then be cloned into an MCS [1]. However, the method requires several consecutive PCR reactions which increase the risk of PCR errors in the final DNA fragment. This complicates or limits the possibilities for making complex constructs where several DNA fragments are combined.

These short-comings are obviated with the USER™ technology. Uracil specific excision reaction (USER™) is a commercial method (New England Biolabs) where custom 3' overhangs, usually of a size of around 10 nucleotides, are generated on DNA fragments from PCR reactions using primers that contain a 2'-deoxyuridine as a substitute of a 2'-deoxythymidine. Fragments can be annealed specifically together and/or into restriction digest-linearized vectors [2]. The overhangs are generated by a 20 minute reaction of a mix of the two enzymes uracil DNA glycosylase (UDG) and the DNA glycosylase-lyase Endonuclease VIII and assembled constructs can be cloned directly by uptake into chemically compentent *Escherichia coli *without the need for a DNA ligation step or DNA purification. The method was recently improved by Nour-Eldin *et al*. [3] with the identification of a commercially available *Pyrococus furiosis *(Pfu) DNA polymerase with proof-reading activity which can read through DNA templates containing deoxyuridine thereby avoiding PCR-introduced nucleotide errors generated by non-proofreading polymerases [3]. Due to the high specificity of the long customly designed 3' overhangs, several DNA fragments can be cloned into a USER™ cassette in one single reaction [4] or multiple fragments can be cloned specifically into different USER™ cassettes in the same vector in a single reaction [5]. This opens a possibility for time-efficient cloning of complex constructs.

The cereal grain is one of the major targets for transformation due to its commercial importance. Also, the grain is an attractive experimental system due to the presence of a number of distinctly different tissues such as pericarp, testa, aleurone, endosperm and embryo with separate functionalities and metabolism that changes during the course of grain filling. Each of these tissues contain cellular compartments of major importance for the proper functioning and nutritional value of the grain such as the starch accumulating amyloplast of the endosperm or the storage vacuoles of the aleurone and embryo. Equally relevant are tissues such as transfer cells that ensure the proper transfer of nutrients from the maternal tissues to the developing grain. Constructs for transformation of the cereal grain will therefore typically contain a promoter that ensures temporal and tissue specific expression as well as targeting signals that confer proper targeting to sub-cellular compartments or the apoplast.

In the present study the USER™ method was combined with assembly PCR for engineering a collection of binary plasmids for *Agrobacterium*-mediated and/or biolistic transformation of cereals. The collection consists of vectors with endosperm-, transfer cell or aleurone cell specific promoters combined with sequences encoding N-terminal transfer peptides for amyloplast targeting and N-terminal signal peptides for ER targeting of the transgenic protein. The vectors were tested by *Agrobacterium*-mediated transformation of barley and transient expression in developing endosperm cells by biolistics to demonstrate the specificity of their expression and targeting of the transgenic protein.

## Results

### Constructing USER™ vectors from existing plasmids

**Figure 1:**
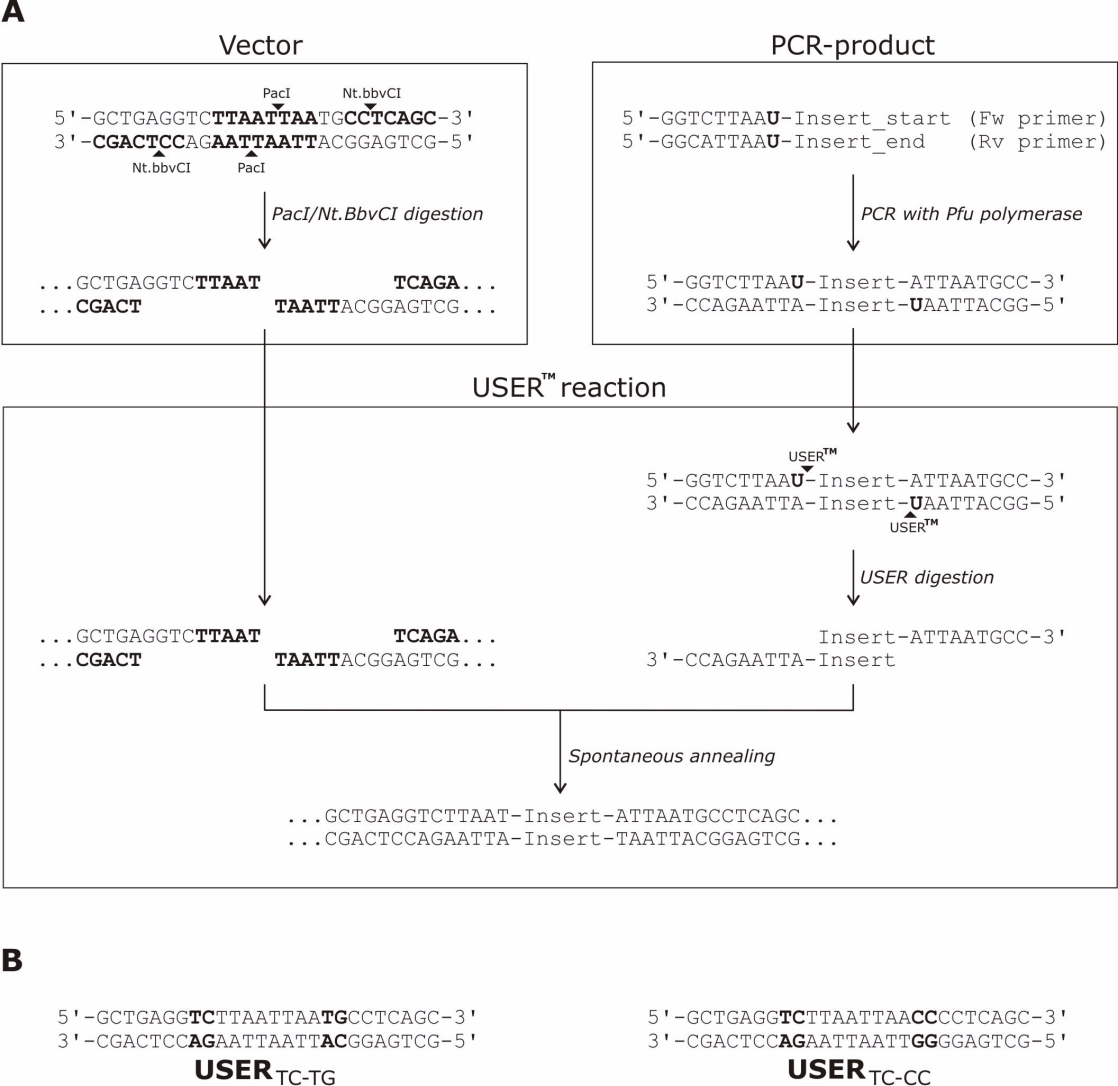
**Overview of the USER™ reaction**. **A**. The vector carrying the USER™ cassette is digested simultaneously with the restriction enzyme *PacI *and the nicking enzyme *Nt.BbvCI*, which creates 9 nt long 3' overhangs. The insert, which is to be fused with the vector, is amplified by PCR using standard PCR primers for the insert, but with the addition of 9 nt complementary to the USER™ site, including a uracil base. In the USER™ reaction USER ™ enzymes remove the uracil creating 9 nt long 5' overhangs complementary to the overhangs of the digested vector. Spontaneous annealing will then result in the desired fusion of the PCR product and vector. Bases in bold indicates the recognition sites of the relevant enzymes, and the arrowheads indicates the site of cleavage. **B**. The two USER™ cassettes designed and used in this study, the USER_TC-TG _and the USER_TC-CC_.

Single or multiple PCR products with long 3' overhangs generated by the uracil specific excision reaction can be inserted into linearized vectors with a compatible USER™ cassette to form a stable plasmid, which can be used to transform *E. coli *without prior ligation (Fig. 1A). This requires that a complementary long 3' overhang is generated from the USER™ cassette when the vector is linearized. This can be achieved by the combined digest of the vector with a restriction enzyme having a very rare and long recognition sequence and a nicking enzyme (cutting only one of the two DNA backbone strands). The nicking enzyme recognition sequence and the rare restriction enzyme recognition sequence are separated by a few nucleotides. The combination of these few nucleotides defines the specificity of each generated 3' overhang. In this study we designed two USER™ sites (USER_TC-TG _and USER_TC-CC_, Fig. 1B) with a spacer of 2 nucleotides which results in 4^2 ^= 16 different types of overhangs. Similarly, 3 nucleotides would have given 4^3 ^= 64 different types of overhangs. We selected a USER™ design based on the restriction enzyme *PacI *with its palindromic recognition site 5'-TTAATTAA-3' and the nicking enzyme *Nt.BbvCI *with its non-palindromic recognition site 5'-GCTGAGG-3'.

**Figure 2:**
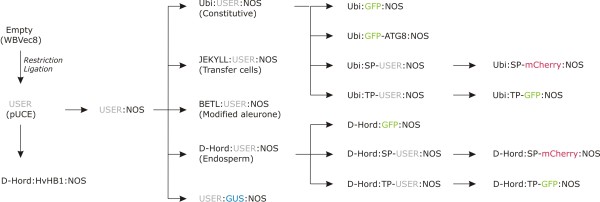
**Overview of pUCE vectors**. A USER_TC-TG _site was introduced in the plasmid pWBVec8 to generate pUCE as described in the text. pUCE_D-Hord:HvHB1:NOS _was generated from pUCE in a single step by combining PCR products of D-Hordein promoter, HvHB1 cDNA and NOS. pUCE_UBI:GFP-ATG8:NOS _was generated from pUCE_UBI:USER:NOS _in a single step by combining PCR products of eGFP and ATG8. The remaining vectors were generated by insertion of single PCR products. A USER™ site is lost when a PCR product is inserted. However, it can be reconstituted if included in one of the primers of the PCR reaction.

As a first step a USER_TC-TG _cassette was inserted into the unique restriction enzyme recognition sites *NotI *and *HindIII *in the MCS of a binary vector to generate the vector pUCE as described in the methods section. In addition to the MCS, the T-DNA part of this binary vector contains a hygromycin phosphotransferase selection marker. After this USER™ cassette insertion step the entire vector collection could be generated by USER™ cloning from pUCE (Fig. 2).

### Single step cloning of binary vectors with multiple inserts

It is possible to insert multiple PCR fragments in a single round of a USER™ cloning reaction, and the ordered directional insertion of up to three PCR fragments has been reported to occur with more than 90% of independent plasmid clones having the intended architecture [4]. Therefore it is possible to engineer in a single step a full construct into a binary vector containing just one USER™ cloning site. As a proof of this concept we first constructed a simple single cDNA expression vector - pUCE_D-Hord:HvHB1:NOS _- by bringing together a promoter, a cDNA open-reading-frame (ORF) and a transcription terminator in a single cloning step. We chose a 436 bp long promoter from a D-hordein gene. D-hordein promoters have earlier been demonstrated to direct strong endosperm specific gene expression [6]. As coding region we selected the small (489 bp) open-reading-frame cDNA from barley plant haemoglobin, a gene which is expressed in the endosperm. The transcription terminator was the standard 258 bp nopaline synthase terminator (NOS). Each fragment was synthesized by PCR, the (non-purified) products were mixed with digested purified vector and a single step USER™ cloning reaction was performed in *E. coli*. Nine out of thirty screened clones were found to possess the correct insertions. Plasmids from five positive clones were purified and sequenced. Only one clone had one single nucleotide error. The rest were correct. Transgenic plants were generated by *Agrobacterium*-mediated transformation to test this vector as described in the methods section.

### Stepwise building of vectors containing cassettes

USER™ sites are not restored when a PCR fragment is inserted (Fig. 1A). However, a new USER™ site can be introduced if it is included in the overhang of one of the primers used for the PCR [3]. This method can be used to build vectors stepwise so that gradually more complex vectors are constructed, and is useful in the case where vectors from each step are wanted for individual cloning purposes. We applied this method to build the vectors in Fig. 2. PCR fragments were amplified as described in the methods section. A NOS terminator was inserted into the USER™ site of the pUCE vector, and a USER_TC-TG _site was reconstituted by including it in the forward primer to generate the pUCE_USER:NOS _vector. The promoters from the D-hordein, JEKYLL, BETL and maize ubiquitin gene were inserted into pUCE_USER:NOS _to generate the constructs pUCE_D-Hord:USER:NOS_, pUCE_JEKYLL:USER:NOS_, pUCE_BETL:USER:NOS _and pUCE_Ubi:USER:NOS_. The maize ubiquitin promoter has been used widely as a constitutive promoter of gene expression in monocots [7]. The D-hordein promoter gives specific expression in developing endosperm, and is useful as a promoter for expression of transgenes specifically in the endosperm [8,9]. The JEKYLL promoter is specific for transfer cells in vascular tissues [10], whereas BETL promoters are active specifically in aleurone cells associated with vascular tissue [11].

The bacterial gene β-glucuronidase (GUS) has been used widely as a reporter gene in plants. We constructed a GUS reporter vector pUCE_USER:GUS:NOS _from pUCE_USER:NOS_. To avoid the occurrence of an unwanted start codon in this vector a USER_TT-CC _site was introduced in this vector rather than a USER_TC-TG _site, which was used for the rest of the collection. In general we saw that the success-rate of correct clones was between 60% and 100% for reactions where a single fragment was inserted by USER™ cloning.

### Using assembly PCR to create tags in USER™ cloning

Assembly PCR is a method in which overlapping synthetic oligonucleotides are designed to recombine into a full double stranded DNA fragment. Fragments of up to at least several hundred base-pairs can be generated [12]. We combined this method with USER™ cloning to demonstrate an easy way to add custom designed targeting sequences to cDNA's in a single cloning reaction. The enzyme Granular-Bound Starch Synthase catalyses the synthesis of amylose in amyloplasts in the endosperm of cereal grains. It contains an N-terminal 70 amino acid long transit peptide (TP) which is cleaved off after entry into the amyloplast [13]. DNA encoding this TP was generated by assembly PCR and inserted into pUCE_D-Hord:USER:NOS _and pUCE_Ubi:USER:NOS _to generate the vectors pUCE_D-Hord:TP-USER:NOS _and pUCE_Ubi:TP-USER:NOS_. D-hordein contains an N-terminal signal peptide (SP) that directs this storage protein to the ER whereafter it is transported to the protein storage vacuole. DNA encoding the D-hordein SP was generated by assembly PCR and inserted into pUCE_D-Hord:USER:NOS _and pUCE_Ubi:USER:NOS _to generate the vectors pUCE_D-Hord:SP-USER:NOS _and pUCE_Ubi:SP-USER:NOS_. Subsequently, a set of vectors was generated where the coding region for either enhanced green fluorescent protein (eGFP) or the red fluorescent protein mCHERRY was inserted into the USER™ cloning site. Transgenic plants were generated to test these vectors.

### Adding tags in a single cloning reaction

To demonstrate that constructs for fusion proteins can be generated in a single cloning reaction we cloned a cDNA encoding ATG8 tagged with eGFP. ATG8 is a small protein that has a characteristic localization pattern, which makes it useful for demonstrating eGFP tagging. It binds specifically to the surface of autophagosomes through a phosphatidylethanolamine lipid anchor, which is posttranslationally conjugated to the C-terminal carboxyl group [14]. eGFP fusions of this protein has been used to monitor macroautophagy and formation of autophagosomes in several eukaryotic cells [15] including monocot plants [16]. PCR products of eGFP and ATG8 cDNA were generated using the primers described in the methods section. The two PCR fragments were cloned in a single step into pUCE_Ubi:USER:NOS _to generate the translational fusion vector for eGFP-ATG8 pUCE_Ubi:GFP-ATG8:NOS_. Three out of five clones had the correct inserts. Transgenic plants were generated to test the vector.

### Testing vectors by plant transformation

**Figure 3:**
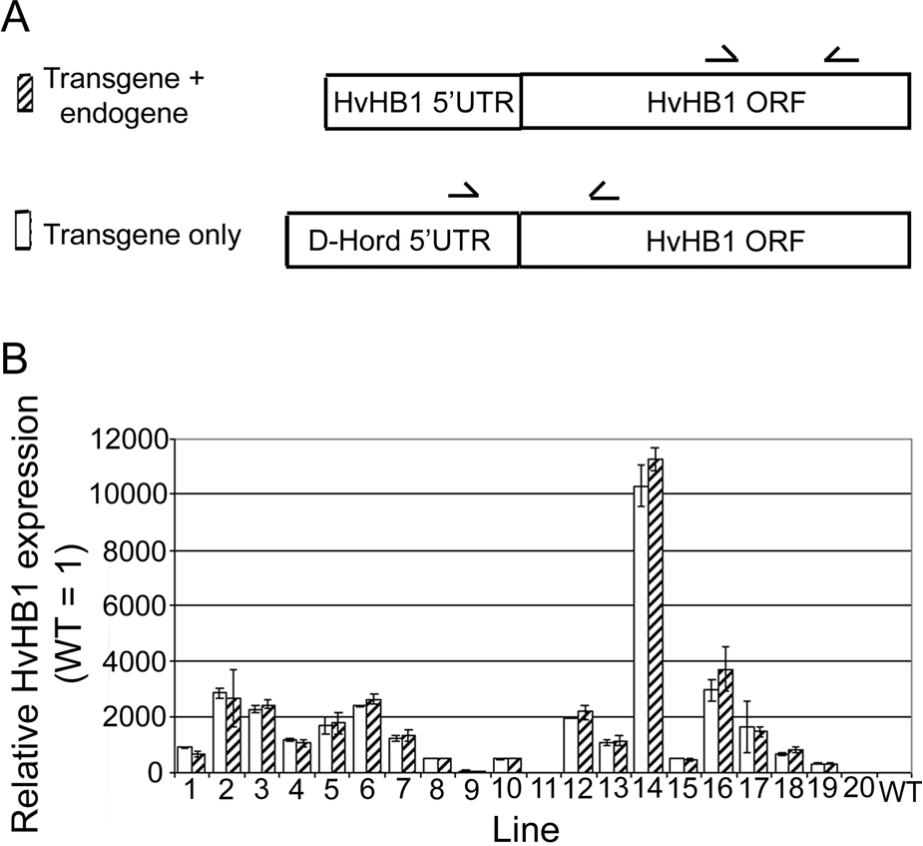
**Transgene over-expression of HvHB1 in endosperm**. Relative level of endogenous and transgenic expression of HvHB1 in the endosperm. **A**, The transgenic cDNA contains the 5' UTR of D-hordein. This was used to design a primer pair that recognizes only transgenic expression of HvHB1 (lower illustration) whereas a primer pair inside the coding region of HvHB1 recognizes both endogenous and transgenic expression of HvHB1 (upper illustration). **B**, Expression in the endosperm was determined by qRT-PCR in wild type barley and 20 putative transgenic lines transformed with pUCE_D-Hord:HvHB1:NOS_. The expression level of endogenous HvHB1 in the wild type plants was defined to be 1.

The simple gene-expression construct pUCE_D-Hord:HvHB1:NOS _designed to over-express the small barley protein HvHB1 was tested by generating transgenic plants. The plants were generated by *Agrobacterium*-mediated transformation of embryonic calli as described by Holme et al. 2006 [17]. A transformation efficiency of 14.5% was observed, which is typical for the protocol. T_1 _seeds were harvested at 15-20 days post anthesis. RNA was purified from endosperm, cDNA first strand synthesised and quantified by quantitative RT-PCR (qRT-PCR) as described in the methods section. The transgenic HvHB1 cDNA contains the 5' UTR from D-hordein. This was used to design two different primer pairs for qRT-PCR: One pair to detect transgenic expression only; and one pair to detect both endogenous gene expression and transgenic over-expression (Fig. 3A). No transgenic HvHB1 expression was detected in the wild type endosperm confirming the specificity of the primers used to detect transgenic expression. A small level of endogenous expression was detected in the wild type endosperm tissue. However, a much higher expression level was detected in endosperm tissues from transgenic lines (Fig. 3B) confirming the functionality of the introduced construct. Transgenic mRNA expression was not detected in leafs, stem and roots of transgenic plants demonstrating the endosperm specific nature of the D-hordein promoter.

**Figure 4:**
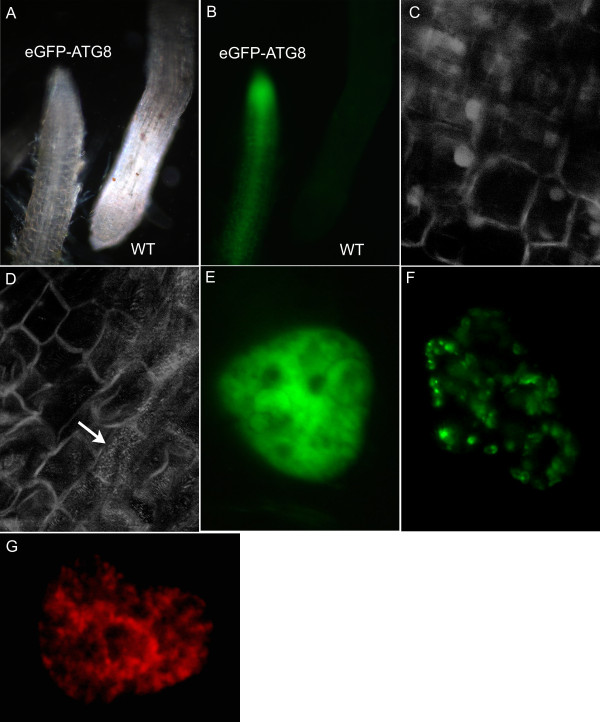
**A-D **Stable transformation (*Agrobacterium*-mediated) - UBI:eGFP-ATG8 roots compared with wild type and UBI:eGFP. **A**, Dark-field. **B**, **e**GFP epifluorescence. **C-D**, CLSM of roots. **C**, UBI:eGFP **D**, UBI:eGFP-ATG8 (Arrow indicates cell with many autophagosomes), **E-H **Transient transformations (biolistic) - endosperm cells with transgenic eGFP or mCHERRY epifluorescence. **E**, D-Hord:eGFP **F**, D-Hord:TP-eGFP **G**, D-Hord:SP-mCHERRY.

The pUCE_Ubi:GFP-ATG8:NOS _vector was used to transform barley plants as described in the methods section. A transformation efficiency of 14% was observed. Roots from T_0 _plants and endosperm from T_1 _seeds were collected from 8 independent lines for analysis. All lines showed a strong eGFP signal in both endosperm and roots when analyzed by epifluorescence as described in the methods section (Fig. 4A &4B). The presence of the eGFP-ATG8 translational fusion protein in roots and endosperm was confirmed by western blotting (not shown). Sub-cellular localization of eGFP-ATG8 in barley roots was studied by confocal laser scanning microscopy (CLSM). This showed localization to sub-cellular spots (Fig. 4D, arrow) with similarity in size and localization to spots that have been identified as autophagosomes in *Arabidopsis *plants expressing eGFP-ATG8 [18]. No such sub-cellular pattern was found in roots of plants expressing eGFP without the ATG8 fusion (Fig. 4C).

The vectors pUCE_D-Hord:GFP:NOS_, pUCE_D-Hord:TP-GFP:NOS_, and pUCE_D-Hord:SP-mCHERRY:NOS _were introduced into endosperm cells by biolistics as described in the methods section and the sub-cellular localization of the transient expression studied by epifluorescence microscopy with filters designed for detection of eGFP or mCHERRY. Cells with expression of eGFP without a signal peptide or a transit peptide showed distribution of eGFP throughout the cytosol (Fig. 4E). In contrast, cells with expression of TP-eGFP (Fig. 4F) showed a much more localized fluorescence in sub-cellular compartments which we interpret to reflect a transport into the plastids. Earlier experiments have demonstrated that the TP from GBSS is an effective tag for targeting of proteins to the plastids [19]. Cells expressing SP-mCHERRY showed targeting to a distinct cellular compartment (Fig. 4G). A signal peptide (SP) directs co-translational targeting of a protein to the ER. Similar localization patterns were seen in endosperm cells expressing the pUCE_UBI:GFP:NOS_, pUCE_UBI:TP-GFP:NOS_, or pUCE_UBI:SP-mCHERRY:NOS _vectors, where expression was driven by an ubiquitin promoter instead of a D-hordein promoter (not shown).

## Discussion

We present here the engineering of a collection of vectors intended for transformation of cereals made by application of the USER™ cloning method [3,4]. It is shown that this method offers a fast way of cloning of unpurified PCR products circumventing problems with multiple cloning sites and ligation of short overhangs. In addition; several PCR products can be cloned into the same site in a specific order in one single cloning step. Earlier problems with stalling of the polymerase by the presence of dUTP in the primers a high error rate during the PCR reaction appears to be solved [3]. According to the manufacturer the PfuTurbo Cx hotstart DNA polymerase, which is used for this purpose, has a rather low error rate (1.3 × 10^-6 ^mutations per base per duplication) and can amplify 1 kb PCR products with 97.4% of the product error free (PfuTurbo Cx Hotstart DNA Polymerase INSTRUCTION MANUAL Catalog #600410, #600412, and #600414, Stratagene). This means that cloning of 5 kb amplification product will give 0.974^5 ^≈ 88% error free clones. The PCR part is however often done on existing plasmids, which gives the opportunity of very precisely optimizing it for a low amount of amplification cycles (15-20 cycles) decreasing the risk of polymerase errors. Mixing the Pfu Cx polymerase with other Pfu polymerases with lower error rates may be a possibility for optimizing the PCR step further.

The currently very popular technique Gateway^® ^cloning (Invitrogen) similarly offers an alternative to traditional DNA recombination by restriction endonuclease and ligases when engineering binary vectors. However, compared to this technique engineering of binary vectors by USER™ cloning of PCR products offers some advantages: The cloning of a PCR product by Gateway^® ^cloning requires the use of two steps -- first cloning into a donor vector then cloning into a destination vector. In contrast, USER™ cloning requires one step and multiple PCR products can be inserted simultaneously in a single cloning step. A tabulated comparison of the different cloning techniques is shown in table 1.

**Table 1:** Comparison of different cloning techniques

	USER™ cloning	Cloning by ligation	GATEWAY^® ^cloning
Requires PCR cleanup when cloning PCR fragments	No	Yes	No^a^

Fragments are allowed to contain restriction enzyme recognition sites	Yes	No^b^	Yes

Number of individual PCR fragments that can be cloned simultaneously in one step	> = 3^c^	1	1^d^

Steps required for cloning into a binary destination vector	1	1 - 2^e^	2^f^

Time required for ligation/recombination of PCR product(s)	40 minutes	1 - 16 hours^g^	1 - 16 hours^h^

The technique allows to make constructs without footprints between assembled fragments	Yes^i^	-	No^d^

Length of overhang in primers used for cloning of PCR products	9 bp	5-10 bp^j^	> 20 bp

a) Cloning may be done without PCR cleanup, however the manufacturer recommends cleaning PCR products before the recombination reaction

b) Fragments cannot contain recognition sites for the restriction enzymes used in the cloning

c) We have demonstrated the simultaneous cloning of three PCR fragments. More fragments may be possible

d) Simultaneous recombination of multiple entry clones may be used to clone up to four fragments into an expression clone by MultiSite GATEWAY^® ^cloning. However, this requires two cloning steps and leaves a footprint from the recombination sites between the fragments.

e) Depends on the vector system

f) GATEWAY^® ^cloning requires that PCR products are cloned into an entry clone before it can be recombined into a destination vector

g) Most protocols recommend ligation for 1-2 hours a room temperature or overnight at lower temperatures

h) Recombination time varies from 1-2 hours to overnight or longer depending on the size of the PCR fragment

i) Multiple fragments cloned in a single step do not have footprints between them. However, sequential USERï¿½ cloning will leave a footprint between fragments.

j) The length depends on what restriction enzymes are used

A toolbox for engineering RNAi constructs for silencing of single genes in cereals already exists: pSTARTGATE http://www.pi.csiro.au/rnai/vectors.htm. USER™ cloning could be used together with this technique to combine PCR products from multiple genes in one cloning reaction to generate an RNAi hairpin construct that would target multiple genes. Such chimeric hairpins have been demonstrated to be effective for simultaneous silencing of several genes [20].

The ability to insert multiple DNA fragments into a USER™ cloning site either in a single cloning step or by consecutive steps offers an opportunity to engineer very complicated constructs. Multiple genes each with their own promoter and transcription terminator may be combined in a single T-DNA, or coding regions for proteins with several domains of different origin and/or different internal or terminal tags may be put together.

## Conclusions

Binary vectors can be engineered by the use of the USER™ cloning system as a time-efficient alternative to existing cloning methods. We constructed a collection of binary vectors for cereal transformation and demonstrated their use as a resource for transformation of barley plants. A number of experiments in which the pUCE toolbox is currently being used to develop transgenic cereals for various purposes are listed in Additional file 1, Table S1.

## Methods

### Insertion of a USER™ cassette

The binary vector pWBVec8 [21,22] was used as a basis for generation of the UCE vector collection. 0.5 μg of the plasmid was digested with the FastDigest™ restriction enzymes *NotI *and *HindIII *(Fermentas Life Sciences) for 10 min in a volume of 13.5 μl. The enzymes were heat inactivated at 80°C for 10 min and after cooling on ice the DNA was precipitated by EtOH (2 μl 3 M sodium acetate pH 4.8, 60 μL 96% EtOH, 5 min centrifugation 14 000 RPM/4°C, wash 70% EtOH, dry). Two customly designed complementary oligos (HPLC grade, Eurofins MWG Operon) USER-F (5'-AGCTT**GCTGAGG**TC**TTAATTAA**TG**CCTCAGC**-3') and USER-R (5'-GGCC**GCTGAGG**CA**TTAATTAA**GA**CCTCAGC**A-3') with overhangs for the *NotI *and *HindIII *were mixed at a concentration of 0.1 pmol/μl. Fourteen μl was used for ligation into the vector by 1 μl of T4 DNA ligase (Invitrogen) in a total volume of 20 μl buffer (supplied by the manufacturer) for 4.5 hours. The construct was cloned in Max Efficiency DH5α chemically competent *E.coli *(Cat. No 18258-012, Invitrogen) as described by the manufacturer. Plasmids were purified using a mini-prep plasmid kit (5Prime, Gaithersburg, Germany) as described by the manufacturer. Positive clones were identified by digest with the enzymes *PacI *and *PstI*, and the correct DNA sequence in and around the insert was confirmed by direct DNA sequencing (Custom DNA sequencing, MWG Eurofins). Five out of six tested clones were positive.

### Generation of single strand DNA overhangs in vectors containing a USER™ site

Vectors containing a USER™ site were made ready for cloning by digesting 5 μg of the vector with 6 μl of the enzyme *PacI *(New England Biolabs) for 15 hours at 37°C in a total volume of 200 μl, followed by 2 hours of digest with freshly added 2 μl *PacI *and 3 μl *Nt.BbvCI *(New England Biolabs) for 2 hours at 37°C. The enzymes were then inactivated by incubating at 80°C for 20 minutes and the plasmid vector was purified in 40 μl 10 mM Tris-HCl, pH 7.5 buffer by using a NucleoSpin™ kit (Macherey-Nagel, Düren, Germany) according to the manufacturers instruction. The purification efficiency was 30-70%.

### Amplification of PCR products for single-step cloning of single or multiple DNA fragments

All PCR reactions of fragments to be cloned into a USER™ site was performed using the *Pyrococus furiosis *(Pfu) DNA polymerase PfuTurbo Cx or PfuTurbo Cx hotstart (Stratagene) in a total volume of 50 μl (5 μl Buffer supplied by the manufacturer, 5 μl dNTP 2 mM each, 5 μl forward primer, 5 μl reverse primer, 26.5 ddH_2_O, 2.5 μl template plasmid DNA 1 ng/μl, 1 μl Cx DNA polymerase). Certain bigger fragments needed double or triple amount of DNA polymerase for PCR to work correctly.

The following primer sets and plasmid DNA templates were used to generate DNA fragments for single insert cloning: NOS, Template: pWBVec8GFP, Primers: 5'GGTCTTAAUTAATGCCTCAGCGATCGTTCAAACATTTGGCAATAAAGTTTCTTAAGATTGAATCCT-3' & 5'-GGCATTAAUCCCGATCTAGTAACATAGATGAC-3'; D-Hordein promoter, Template: Genomic DNA, Primers: 5'-GGTCTTAAUTTCGAGTGCCCGCCGATTTGCCAG-3' & 5'-GGCATTAAUTAAGACCTCAGCTCTCGGTGGACTGTCAATGAA-3'; HvHB1, Template: CA2α Primers: 5'-GGTCTTAAUCGAGATGTCTGCCGCG GAGGGG-3' & 5'-GGCATTAAUCTACTCAGCTGGCTTCATCTC-3'; GFP, Template: pWBVec8GFP, Primers: 5'-GGTCTTAAUATCCATGGTGAG CAAGGGCG-3' & 5'-GGCATTAAUTTACTTGTACAGCTCGTCCATG-3'; mCHERRY, Template: pLIFE51, Primers: 5'-GGTCTTAAUATCCATGGTGAGCAAGGGCGAGG-3' & 5'-GGCATTAAUTTA CTTGTACAGCTCGTCCATGC-3'; JEKYLL promoter, Template: Genomic DNA, Primers: 5'-GGTCTTAAUTAGGGCACGCGTGGTC-3' & 5'-GGCATTAAUTAAGGATCCTTAATTAAGCCTCAGCCCTGCGCGATCGAGCTTGC-3'; BETL4 promoter, Template: Genomic DNA, Primers: 5'-GGTCTTAAUTTGGCACGCGTGGTCGA-3' & 5'-GGCATTAAUTAAGGATCCTTAATTAAGCCTCAGCCCGTTCTATGTTGTGTTGT-3'. The following primer sets and plasmid DNA templates were used to generate DNA fragments for multiple insert cloning: D-Hord:HvHB1:NOS: D-Hordein promoter, Template: Genomic DNA, Primers: 5'-GGTCTTAAUTTCGAGTGCCCGCCGATTTGCCAG-3' & 5'-ATCTCGGTGGACUGTCAATGAA-3' & HvHB1, Template: CA2α [23] Primers: 5'-AGTCCAACCGAGAUGTCTGCCGCGGAGGGGGCCGTC-3' & 5'-AACGATCGTGGAGCUACTCAGCTGGCTTCATCT-3' & NOS, Template: pWBVec8GFP, Primers: 5'-AGCTCCACGATCGTUCAAACATTTGGCAATAAAGTTTC-3' & 5'-GGCATTAAUCCCGATCTAGTAACATAGATGAC-3'; GFP-ATG8: GFP, Template: pWBVec8GFP, Primers: 5'-GGTCTTAAUATCCATGGTGAG CAAGGGCG-3' & 5'-ACCTCCCTUGTACAGCTCGTCCATGC-3' & ATG8, Template: pTOPO-ATG8 (Jan Svensson, unpublished), Primers: 5'-AAGGGAGGU GCCAAGAGCTCGTTCAAGCTG-3' & 5'-GGCATTAAUCTAGAGCGATTCGAGCGATCCAAA G-3'. Due to difficulties in amplifying the full maize ubiquitin-1 promoter two pieces of DNA was amplified by PCR: Template: pWBVec8GFP Primers: 5'-GGTCTTAAUGTTTGACAGCTTATCATCGA-3' & 5'-ATGAACAGAAGUAGAACTACCG-3' and 5'-ACTTCTGTTCAUGTTTGTGTTAGAT-3' & 5'-GGCATTAAUTAAGACCTCAGCCTGCAGAAGTAACACCAAACAAC-3'

Assembly PCR was performed as described by Rydzanicz et al. [10], using the indicated software for design of oligonucleotides. The set of oligonucleotides and primers used to generate DNA fragments for cloning is described in Additional file 1, Table S2 for GBSS Transit Peptide and Additional file 1, Table S3 for D-hordein signal peptide.

### USER™ cloning

USER™ cloning was performed in a total volume of 12 μl (10 μl PCR product, 1 μl USER™ Enzyme mix (New England Biolabs), 1 μl pre-digested USER Vector 50-100 ng/μl). The USER™ Enzyme mix is fully functional in the PCR buffer and the reaction can be performed directly with the unpurified PCR product reaction. The USER reaction mix was incubated for 20 min at 37°C for digestion followed by 20 min at 25°C for annealing of complementary overhangs. 1 μl of the USER™ reaction mix was used to transform 50 μl Max Efficiency DH5α chemically competent *E.coli *(Cat. No 18258-012, Invitrogen) as described by the manufacturer. Plasmid midi prep was performed with a Midi Nucleoband AX kit (Macherey-Nagel, Düren, Germany) according to the manufacturer's description. Positive clones were identified by direct sequencing.

### RNA purification and qRT-PCR

Total RNA from roots, leaves and grain endosperm was purified using a FastRNA^® ^Pro Green kit (MP Biomedicals LLC, France). 3 μg total RNA was used to generate first strand cDNA by using Superscript Reverse Transcriptase II (Invitrogen) and random hexamer oligonucleotide primers in a total volume of 20 μl as described in the manufacturers guidelines. The finished product was diluted 10 times to be ready for qPCR. qPCR was performed in an ABI Prism 7900HT Sequence Detection System. Power SYBR^® ^Green PCR Master mix (Applied Biosystems) was used as a basis for the reaction in a total volume of 10 μl (5 μl PCR mastermix, 1 μl Primer mix 5 μm each primer, 3 μl H_2_0, 1 μl cDNA). The following primer pairs were used to amplify the gene of interest: HvHB1 5'-TCGTCTTCAGCGAGGAGAAG-3' & 5'-GATCTCGAAGATCTTGAGGAAG-3' for detection of wild type (endogene) mRNA expression and 5'-GATCAATTCATTGACAGTCCAC-3' & 5'-TCGCAGGTCATGACGAAGAC-3' for detection of transgenic D-hordein:HvHB1 mRNA expression; Housekeeping gene GADPH 5'-GCTCAAGGGTATCATGGGTTACG-3' & 5'-GCAATTCCACCCTTAGCATCAAAG-3'. Measurements were based on three technical replicates.

### Plant growth, transformation and microscopy

Barley plants (*Hordeum vulgare *cv. Golden Promise) was grown in a controlled growth chamber (12°C-14°C) under a 16 hours day (550 μEinsteins)/8 hours dark cycle, and relative humidity at 90-95%. *Agrobacterium*-mediated transformation of barley embryos was carried out as described by [17]. Transformation of barley endosperm cells was done by biolistics as described before for embryos [24].

Transgenic plants were analyzed by a Leica dissection microscope and a Zeiss Axioplan 2 epiflourescence microscope using the proper filters for detection of eGFP and mCHERRY. CLSM was carried out on a Bio-Rad MRC-1024 Confocal Laser Scanning Microscope.

## Competing interests

The authors declare that they have no competing interests.

## Authors' contributions

KHH constructed the plasmids pUCE, pUCE_USER:NOS_, pUCE_D-Hord:USER:NOS_, pUCE_D-Hord:TP-USER:NOS_, pUCE_UBI:TP-USER:NOS_, pUCE_USER:GUS:NOS_, conducted the testing of the vectors and wrote the manuscript including figure 3 and 4. MWC constructed the plasmids pUCE_UBI:USER:NOS _and pUCE_USER:GUS:NOS_, made the figures 1 and 2 and assisted in the testing of the vectors. MC constructed the plasmids pUCE_D-Hord:SP-USER:NOS _and pUCE_UBI:SP-USER:NOS _and assisted in the testing of the vectors. BT made the plasmids pUCE_JEKYLL:USER:NOS_, pUCE_BETL:USER:NOS_. HBP assisted in the testing of the vectors and cloned the D-hordein promoter. All authors commented on and approved the manuscript.

## Supplementary Material

Additional file 1 **Contains Table S1, Table S2 and Table S3**. Click here for file
