# Supplementary material for: UCE: A uracil excision (USER™)-based toolbox for transformation of cereals
Source: Plant Methods. 2010 Jun 10;6:15. doi: 10.1186/1746-4811-6-15 (PMC2892451; doi:10.1186/1746-4811-6-15)
Supplement: Additional file 1 — Contains Table S1, Table S2 and Table S3. [file 1746-4811-6-15-S1.DOC]

**Table S1:** Experiments using pUCE toolbox for engineering of transgenic plants

| pUCE vector | Insert | Purpose of transgenic plants |
| --- | --- | --- |
| pUCE_USER:GUS_ | Promoter region of HvNAC005 or HvNAC013 | Barley GUS promoter reporter lines of the NAC transcription factors HvNAC005 and HvNAC013 |
| pUCE_JEKYLL:USER:NOS_ | Coding region of HvHMA2 | Over-expression of HvHMA2 – a P-type ATPase efflux transporter in transfer cells for enhanced transport of Zn into grain endosperm cavity to obtain Zn bio-fortified barley grains |
| pUCE_D-hord:TP-USER:NOS_ | Coding region of StGWD | Over-expression of potato glucan water dikinase (StGWD) in barley endosperm amyloplasts for hyper-phosphorylation of starch in grains |
| pUCE_D-hord:TP-USER:NOS_ | Coding region of HvHB1 | Over-expression of barley plant hemoglobin 1 in amyloplasts for accumulation in barley grain endosperm of heme-chelated iron with high bio-availability |
| pUCE_UBI:USER:NOS_ | Coding region of HvHB1 | Ubiquitous over-expression of plant hemoglobin 1 in barley to obtain varieties with enhanced resistance to flooding |

| **Table S2:** Primers and oligonucleotides for assembly PCR of GBSS transit peptide | |
| --- | --- |
| Primers | 5’-GGTCTTAAUcgccatggcggctctggccacg -3’  5’-GGCATTAAUGCTGAGGCATTAATTAAGACCTCAGCGCGCACCACCACGGAGAGGCA-3’ |
| Oligonucleotides | 5’-ATGGCGGCTCTGGCCACGTCCCAGCTCGCCACCTCCGG-3’  5’-CGCCGGAATCTGTCGGTGACGCCGAGGACGGTGCCGGAGGTGGCGAG-3’  5’-ACCGACAGATTCCGGCGTCCAGGTTTTCAGGGCCTCAGGCCCCGGAA-3’  5’-ATAGTCCTCATACCAAGCGCCGCATCCGCTGGGTTCCGGGGCCTGAGGC-3’  5’-GCGCTTGGTATGAGGACTATCGGAGCAAGCGCCGCCCCGAAGCAAAGCC-3’  5’-ACCGCCGGCTCCCGCGGTGCGCTTTCCGGCTTTGCTTCGGGG-3’  5’-ccgcgggagccggcggtgcctctccgtggtggtgcgcgctgtactta-3’  5’-gcaccaccacggagaagaggtatcagtggtaagtacagcgc-3’ |

| **Table S3:** Primers and oligonucleotides for assembly PCR of D-hordein signal peptide | |
| --- | --- |
| Primers | 5’- GGTCTTAAUCGAGATGGCTAAGC -3’  5’- GGCATTAAUGCTGAGGCATTAATT -3’ |
| Oligonucleotides | 5’- GCTCTCACCACCGCTGAACGTGAGATCAA GGGAACAACGGAGGAGCT GAGGT -3’  5’-GGTCTTAATCGAGATGGCTAAGCGGCTGGTCCTCTTTGTGGCG-3’  5’-GGCATTAATGCTGAGGCATTAATTAAGACCTCAGCTCCTCCGTTG-3’  5’-TTCAGCGGTGGTGAGAGCCACGAGGGCGACGATTACCGCCACAAAGAGGACC-3’ |
